# Twisted Epithelial-to-Mesenchymal Transition Promotes Progression of Surviving Bladder Cancer T24 Cells with hTERT-Dysfunction

**DOI:** 10.1371/journal.pone.0027748

**Published:** 2011-11-15

**Authors:** Yan Xue, Lei Li, Dong Zhang, Kaijie Wu, Yule Chen, Jin Zeng, Xinyang Wang, Dalin He

**Affiliations:** Department of Urology, First Affiliated Hospital of Medical School, Xi'an Jiaotong University, Xi'an, Shaanxi, China; Roswell Park Cancer Institute, United States of America

## Abstract

**Background:**

Human cancer cells maintain telomeres to protect cells from senescence through telomerase activity (TA) or alternative lengthening of telomeres (ALT) in different cell types. Moreover, cellular senescence can be bypassed by Epithelial-to-mesenchymal transition (EMT) during cancer progression in diverse solid tumors. However, it has not been elucidated the characteristics of telomere maintenance and progression ability after long-term culture in bladder cancer T24 cells with hTERT dysfunction.

**Methodology/Principal Findings:**

In this study, by using a dominant negative mutant human telomerase reverse transcriptase (hTERT) vector to inhibit TA in bladder cancer T24 cells, we observed the appearance of long phenotype of telomere length and the ALT-associated PML body (APB) complex after the 27^th^ passage, indicating the occurrence of ALT-like pathway in surviving T24/DN868A cells with telomerase inhibition. Meanwhile, telomerase inhibition resulted in significant EMT as shown by change in cellular morphology concomitant with variation of EMT markers. Consistently, the surviving T24/DN868A cells showed increased progression ability *in vitro* and *in vivo*. In addition, we found Twist was activated to mediate EMT in surviving T24/DN868A samples.

**Conclusions/Significance:**

Taken together, our findings indicate that bladder cancer T24 cells may undergo the telomerase-to-ALT-like conversion and promote cancer progression at advanced stages through promoting EMT, thus providing novel possible insight into the mechanism of resistance to telomerase inhibitors in cancer treatment.

## Introduction

The stabilization of telomere is critical for the infinite cellular proliferation which is necessary for tumor formation [1]. At least two mechanisms have been identified to maintain telomere homeostasis in cells *in vivo* as well as *in vitro*. First, most carcinoma cells utilize telomerase, a multi-subunit cellular ribonucleoprotein enzyme (referred to as a telomerase pathway), to add telomeric repeats onto chromosome ends [2]. Telomerase is composed of an RNA subunit, telomerase-associated protein 1, and the catalyst telomerase reverse transcriptase (hTERT), which is a limiting component in regulating the activity of telomerase [3]. Second, sarcoma cells utilize a telomerase-independent mechanism termed alternative lengthening of telomeres (i.e., ALT pathway) to maintain chromosome termini [4]. The onset of ALT is determined by the appearance of long, heterogeneous telomere length, and the appearance of ALT-associated promyelocytic leukemia bodies (APBs) in about 10% of interphase nuclei [5].

Previous reports have indicated that telomerase and the ALT mechanism could coexist in some tumors [4], [6]. Furthermore, a reversible conversion of telomerase-positive to telomerase-negative cells has been reported in cases of human papillomavirus type 16 E6-immortalized fibroblasts [7]. In view of the importance of telomerase in cell immortalization and lack of telomerase expression in most normal somatic cells, telomerase inhibitors held considerable promise for cancer therapy in the past [8]. But the possibility of activation of ALT which is resistant to telomerase inhibitors complicates this situation [9]. It is well known that ALT confers properties different from telomerase on cancer malignancy. In the case of glioblastoma multiforme, ALT correlated with a better patient prognosis [10], whereas a poor prognosis was detected in patients with liposarcoma [11]. The reason for the difference of cancer malignancy remains elusive.

In addition to telomere maintenance, the acquisition of a fully malignant phenotype also includes the requirement of metastasis. Epithelial-to-mesenchymal transition (EMT) plays an important role in the progression of primary tumors toward to metastases [12]. Recent observations suggest that EMT and cell senescence are crossed in cancer progression [13]. Several distinct transcription factors, which have been found to be capable of inducing EMT programs, such as Twist and ZEB1, can also protect cells from senescence induced by the *ras* oncogene [14], [15].

In this work, we used a dominant negative mutant of hTERT to constitutively inactivate telomerase activity (TA) in bladder cancer T24 cells. Our data show that long telomere length and APBs complex without the up-regulation of TA can occur during long-term culture in bladder cancer cells *in vitro*. Strikingly, a cross between EMT and telomere maintenance pathway was observed concomitantly with increased progression ability. Furthermore, Twist was activated to induce EMT. In summary, we describe a novel mechanism in the acquisition of invasive features and telomere homeostasis after telomerase inhibition in bladder cancer T24 cells, thus providing insight into drug resistance after telomerase inhibition in bladder cancer.

## Methods

### Ethics Statement

All experiments on the animals conform to the *Guidelines of Animal Care* of Xi'an Jiaotong University and approved by the Ethical Review Board (ERB) committee (The First Affiliated Hospital of Medical College, Xi'an Jiaotong University, China), and the approval ID of the ethic board is SCXK2007-0005.

### Antibodies

Antibodies against PML, TRF2, N-cad, Vimentin, Cytokeratin-18, 19 (CK-18, CK-19), Matrix metalloproteinases-2 (MMP-2) and Twist were from Santa Cruz Biotechnology Inc. (Santa Cruz, CA).

### Cell culture

The human bladder cancer cell line T24 and osteosarcoma cell line U_2_OS were cultured in Dulbecco's modified Eagle's Medium (GIBCO, Grand Island, NY) supplemented with 10% (v/v) fetal bovine serum (FBS, Sijiqing, Hangzhou, China) at 37°C with 5% CO_2_ in a humidified incubator.

### Establishment of hTERT^DN868A^ Stable cells and transient Twist transfection

The dominant negative mutant construct of hTERT (PCI-neo-hTERT^DN868A^) was verified by sequencing before transfection into cultured T24 cells. siRNA for Twist were designed and synthesized by Invitrogen (Shanghai, China). The sequence of siTwist was: sense 5′-CACCGGACAAGCUGAGCAAGAUUCACGAAUGAAUCU UGCUCAGCUUGUCC-3′; antisense 5′-AAAAGGACAAGCUGAGCAAGAUUCA UUCGUGAAUCUUGCUCAGCUUGUCC-3′. Transfection was carried out using LipofectAMINE 2000 (Invitrogen, Inc., Gaithersburg, VA) according to the manufacturer's protocol. Stable transfectants were selected with 300 ug/ml G418 (Merck, Darmstadt, Germany).

### Telomerase Activity Assay

Telomerase activity was measured using a TRAP-PCR-ELISA assay kit following the manufacturer's instructions (Roche, Basel, Switzerland). Briefly, 2×10^5^ cells were suspended with lysis buffer, and an equal amount of nuclear supernatant was used as a TRAP template for PCR reaction. The reaction mixture was incubated at 25°C for 30 minutes, and then PCR was amplified for 35 cycles at 94°C for 30 s seconds and 59°C for 60 seconds. Then product was mixed with a hybridization solution and incubated at 37°C for 1 hour, followed by washing and incubating for 30 minutes. It was then chromogenized and the absorbance was detected.

### TRF analysis

DNA was extracted using a DNA isolation kit (Roche, Basel, Switzerland) and aliquots (2 µg) digested 2 hours with the restriction enzymes *Rsa*I and *Hinf*I. Digested DNA fragments were separated in 0.8% agarose gels and blotted onto positively charged nylon membranes. Telomere restriction fragments were revealed through hybridization with DIG-labeled telomere probe and chemiluminescence detection.

### Q-FISH for Cytogenetic Analysis

Chromosomes from colcemid arrest cells were prepared and followed a standard Q-FISH technique [16] using the Telomere PNA FISH/FITC kit (Dako Cytomation, USA). In brief, detached cells were treated with 50 mmol/L KCl and fixed using 3∶1 methanol/glacial acetic acid; then slides were prepared. Telomere was detected using an FITC-conjugated peptide nucleic acid (PNA) probe and counterstained with DAPI. Digital images were captured under confocal microscopy at 60× or 120× objectives.

### Double Immunofluorescence

Cells were grown on glass coverslips and fixed in 4% paraformaldehyde (PFA). After washing with PBS, the cells were permeabilized using 0.1% Triton X-100 solution and blocked with 10% donkey serum. Following incubation with the primary antibodies PML and TRF2, they were incubated with the fluorescently labeled secondary antibodies Alexa Fluor 488, Alexa Fluor 555/568 (invitrogen). Images were collected under confocal microscopy and processed using Adobe Photoshop 7.0.

### Western blotting

Western blotting was carried out as described previously [17]. Briefly, samples were analyzed by 12% SDS-PAGE, transferred to nitrocellulose membranes, and probed with the primary antibodies and secondary antibodies coupled to horseradish peroxidase, then detected by the ECL chemiluminescent detection system (Amersham, Piscataway, NJ).

### Invasion and Migration Assay

The invasion and migration capability of T24 cells were determined by the Transwell assay. For the invasion assay, 50 µL of Matrigel (Sigma, Inc.) was applied to 8 µm-pore-size polycarbonate membrane filters (Corning, Inc., Corning, NY), the bottom chamber of the apparatus containing DMEM with 20% FBS. 5×10^3^ T24, T24/PCI, and the 32^nd^ passage of T24/DN868A (defined as surviving T24/DN868A in the following study) cells were seeded with a serum-free medium, and then incubated for 48 hours at 37°C. Following incubation, the invaded cells attached to the lower surface of the membrane were fixed by 4% paraformaldehyde and stained with Giemsa. Cell numbers were counted in three randomly chosen microscopic fields (10×) per membrane. The migration assay was carried out as described for the invasion assay, with no coating of Matrigel.

### Adhesion assay

Matrigel was dilute with serum-free DMEM at 1∶20 and coated onto bottom of 96-well plate. 1×10^4^ cells were harvest and seeded in 96-well plate with serum-free medium and allowed to attach to Matrigel. After 6 h, medium with unattached cells was removed and MTT were added. 4 hrs of incubation later, DMSO was added to solubilize the formazan crystals and the absorbance (O.D.) at 590 nm was measured.

### Soft Agar Colony Formation Assay

The colony formation assay was performed as described previously [18]. Briefly, a total of 500 cells were suspended in 2 mL DMEM containing 0.3% low melting agar and overlying the 2 mL base layer consisted of 2× DMEM and 1.2% agar (1∶1 mixed) in six-well plates. After incubation at 37°C with 5% CO_2_ in a humidified incubator for 14 d, colonies more than 50 cells were counted.

### Tumorigenicity Assay *in Vivo*

Four to six weeks old nude athymic BALB/C mice (Shanghai Experimental Animal Center, Shanghai, China) were used for the tumorigenicity assay. Cells were harvested, washed, and resuspended in serum-free DMEM at the concentration of 1×10^7^ cells/ml, and 1×10^6^ cells were injected subcutaneously. The mice were sacrificed after 8 weeks to measure the tumor weight. Immunohistochemical staining was carried out as described previously [19].

### Statistical Analysis

All data analyses were done by the software SPSS 13.0 for Windows. *P*<0.05 was regarded as the threshold value for statistical significance.

## Results

### Telomerase activity in T24 cell clone expressing dominant-negative (DN) hTERT

**Figure 1:**
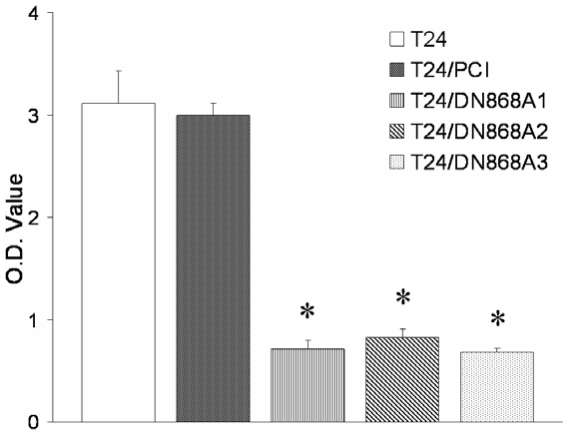
Telomerase activity in T24 cells with different transfectants. Telomerase activity (TA) in T24 cells transfected with various constructs was determined as described in Materials and Methods (* *P*<0.05).

To constitutively inactivate hTERT, the human bladder cancer cell line T24 with high TA was transfected with a plasmid encoding DN-hTERT or empty vector PCI, and individual cell clones were isolated by G418 screening for 7 weeks. TRAP-PCR-ELISA analysis demonstrated that in T24/DN868A cells at the 3^rd^ passage, TA was sharply reduced compared to the parental cells (Fig. 1). Three clones with decreased TA were screened and designated as T24/DN868A (M1∼3). The empty vector-transfected control cells were designated as T24/PCI.

### Telomere length dynamics in T24 cell clone expressing DN hTERT

**Figure 2:**
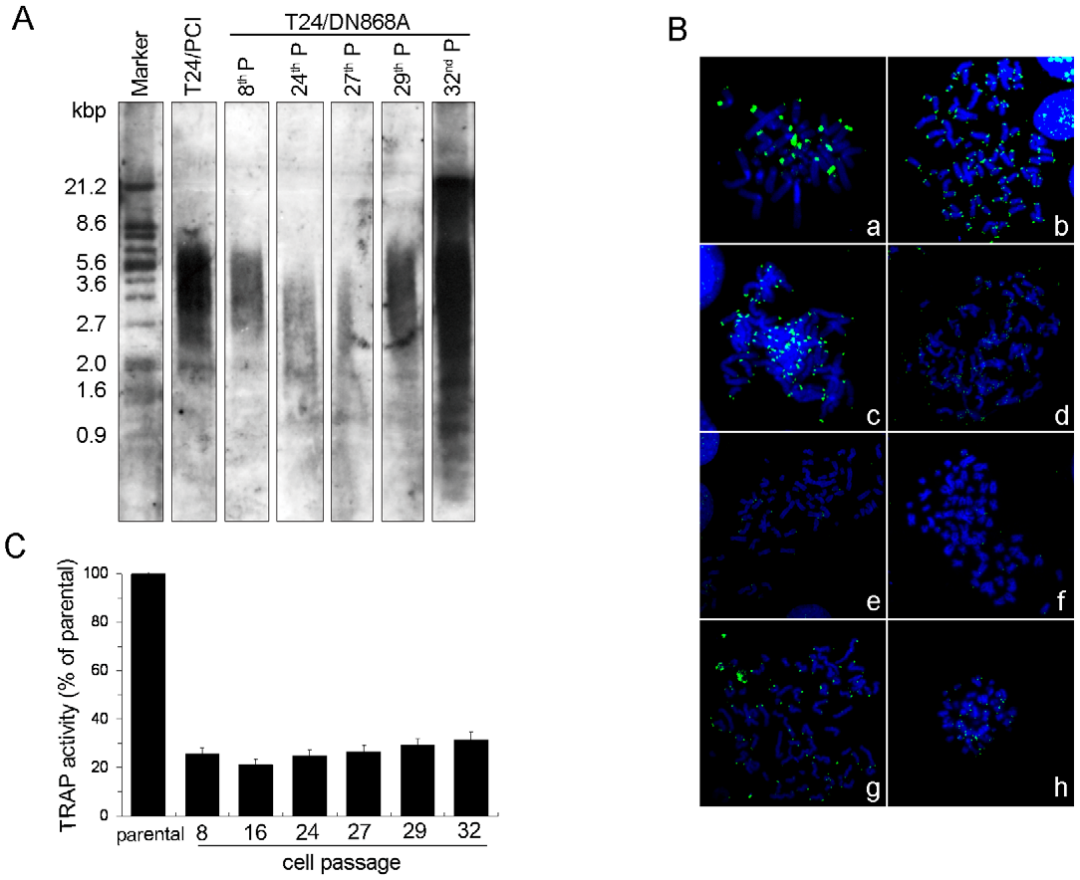
Telomere length dynamics in T24 cells with different transfectants. A. TRF analysis of T24/DN868A cell clone with different passage. DNA was extracted and performed using gel electrophoresis as described in Materials and Methods. B. Telomere length analysis by Q-FISH on individual metaphase chromosomes from parental and transformed T24 subclones of different passages using FITC-labeled PNA telomere probes (green) and counterstained with DAPI (blue). The intensity of green signals corresponds to the length of telomeres. a) U_2_OS cells; b) T24; c) T24/PCI; d∼f) T24/DN868A subclones of 8^th^, 24^th^, and 27^th^ passages, respectively; g and h) T24/DN868A subclones of the 29^th^ and 32^nd^ passage, respectively (120×). C. TA of cells at each time point when telomere length analysis was done. Histograms are representative of the TA in T24/DN868A versus parental cell line.

To visualize telomere length of T24/DN868A cells at different passage from earlier to later, TRF analysis was followed in these three isolated clones. As shown in Fig. 2A, the median telomere length was ∼4.3 kb at 6^th^ passage of T24/DN868A-M1 and continuous telomere shortening was observed until the 27^th^ passage. This phenomenon was accompanied by a reduction of the proliferation rate from 1 passage/2.5 per day to 1 passage/6 per day. After the 27^th^ passage, telomere elongation was appeared again. A median length of ∼6 kb with the occurrence of an additional high molecular weight band at ∼21 kb was displayed at 29^th^ passage. In addition, the proliferation rate of T24/DN868A cells at this stage was increased to 1 passage/3 per day.

Furthermore, we stained the metaphasic chromosomes of T24/DN868A cells with a telomeric PNA probe, and compared them with those parental or control cells at the same passage. The osteosarcoma cell line U_2_OS was used as an ALT-positive control (Fig. 2B a) [20]. Fig. 2B shows Q-FISH analysis of one of the clones. The very intense green signals at the end of chromosome corresponded to the long telomeres. A detectable telomere FISH signal could be seen at almost every chromosome end and in the majority of nuclei in a different passage of parental T24 cells or control cells T24/PCI; furthermore, the intensity of these signals in both two cells was consistent (Fig. 2B b∼c). After telomerase inhibition, although the intensity of the green signal at each end of chromosome or in the nuclei was consistent with the earlier passage of T24/DN868A, the telomere signals decreased gradually with each round of cell division until the 27^th^ passage, at which point, the overall intensity of the fluorescent signal was lowest (Fig. 2B d∼f). After this passage, signals with different intensity representing heterogeneous telomere length were seen at the end of some chromosomes in T24/DN868A cells (Fig. 2B g∼h). For the other two tested T24/DN868A clones expressing DN hTERT, both showed strong telomerase inhibition, but only one (T24/DN868A-M3) displayed telomere length fluctuations as Fig. 2A and B, while the other one (T24/DN868A-M2) went to cell death and loss of the culture at the 16^th^ passage.

To analyze the nature of telomere elongation events, we examined TA at each time point when Q-FISH was done in these two viable cell clones. Inhibition of TA by DN hTERT was robust and consecutive (up to the 32^nd^ passage), and the same low level of TA was observed consecutively in T24/DN868A cells (Fig. 2C). It is therefore unlikely that the remaining telomerase is the cause for the observed telomere elongation.

### APBs status in T24 cell clone expressing DN hTERT

**Figure 3:**
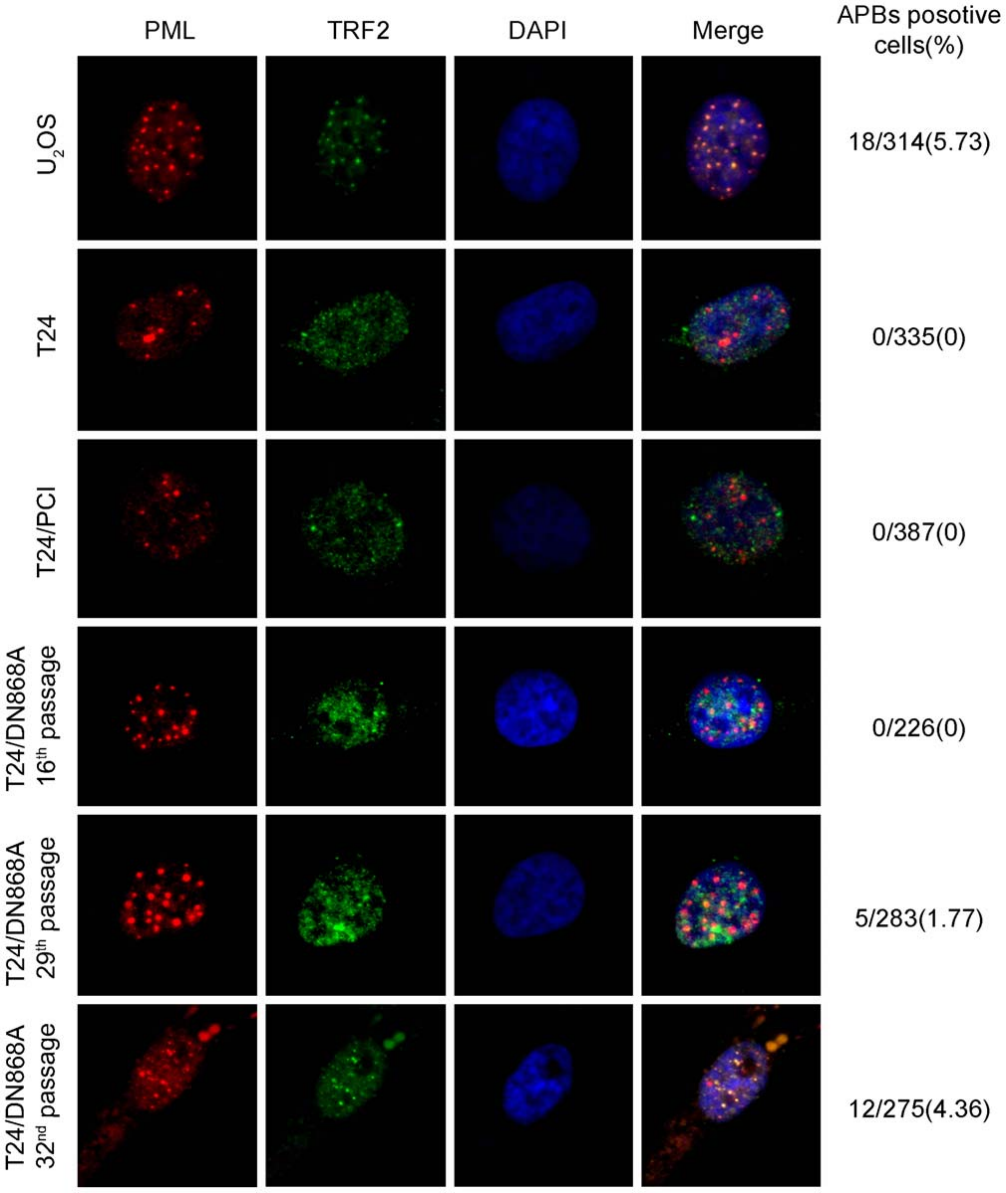
APBs status in different passages of hTERT-inactivated T24 cells. Double immunofluorescence analysis for APBs in hTERT-inactivated T24 at different passages. Red spots represent PML, green spots represent TRF2, and yellow sparkles indicate the colocalizaiton of PML with TRF2. APBs was absent in the T24, T24/PCI, and earlier passage of T24/DN868A cells but present in T24/DN868A cells at the 29^th^ and 32^nd^ passage (60×).

APBs complex is a subset of promyelocytic leukemia (PML) bodies containing telomeric DNA and telomere-binding proteins such as TRF1 and TRF2. APBs may play an integral role in the ALT mechanism, and are not found in mortal or telomerase-positive cells [21]. Therefore, we detected the presence of APBs by co-localization of PML with TRF2 in various T24 cells at different passages compared with the osteosarcoma cell line U_2_OS. As shown in Fig. 3, the co-localization of PML with TRF2 could not be detected in the parental T24, T24/PCI cells, or earlier passages of T24/DN868A cells. However, in the 29^th^ passage of T24/DN868A cells, the yellow sparkles representing APBs were clearly observed in some T24/DN868A cells. Moreover, an increase in the overall number of APBs was observed in the 32^nd^ passage of T24/DN868A cells. These results strongly indicate the presence of an ALT-like mechanism of telomere stabilization in the subclone of T24/DN868A cells from the 29^th^ passage. To distinguish the late passage of T24/DN868A cells with characteristics of ALT-like from earlier passage, all the T24/DN868A cells after the 29^th^ passage were called as surviving T24/DN868A cells. One of each surviving cell clone at the 32^nd^ passage was randomly used for the following study.

### Induction of EMT in T24 cells with telomerase inhibition

**Figure 4:**
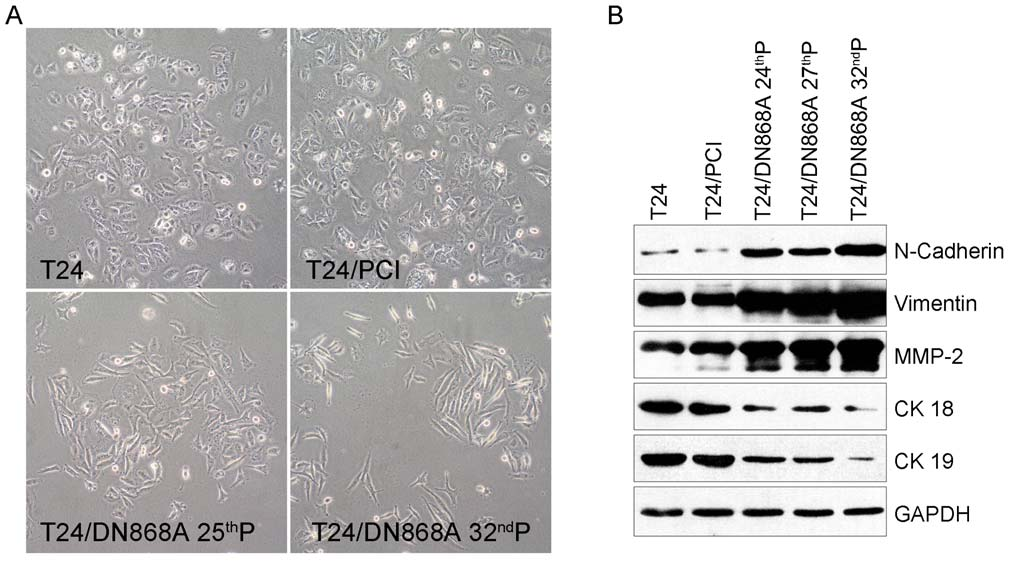
Cell morphology and analysis of EMT markers. A. cell morphology of T24, T24/PCI and T24/DN868A cells were observed under a phase-contrast microscopy (20×). Cell morphology changed from polygonal, epithelial structure (upper) to spindle-like, mesenchymal structure (down). B. Western blot analysis of N-Cadherin, Vimentin, MMP-2, CK18, and CK19 levels in T24, T24/PCI, and different passage of T24/DN868A cells. GAPDH was used as a loading control.

T24 is an E-cadherin deficient cell line which yet exhibits an epithelial-like morphology [22]. During long term culture, we found that T24 cells with telomerase inhibition gradually exhibited a spindle-shaped, more elongated morphology and loose intercellular junctions from the 25^th^ passage while T24 or T24/PCI cells were still polyhedral and grew in a tightly connected manner, which suggested that the cells may have undergone EMT (Fig. 4A). To test this, detailed molecular characterization of EMT markers of T24, T24/PCI, and different passage of T24/DN868A cells (including 25^th^, 27^th^, and 32^nd^ passage) were detected by Western blot. Indeed, we observed the loss of epithelial markers including CK18 and CK19 and increased levels of mesenchymal markers and invasion-related proteins such as N-cadherin, Vimentin, and MMP-2 in T24/DN868A cells from 25^th^ passage (Fig. 4B).

### Decreased adhesion, increased migration, invasion, and colony formation of surviving T24/DN868A cells *in vitro*

**Figure 5:**
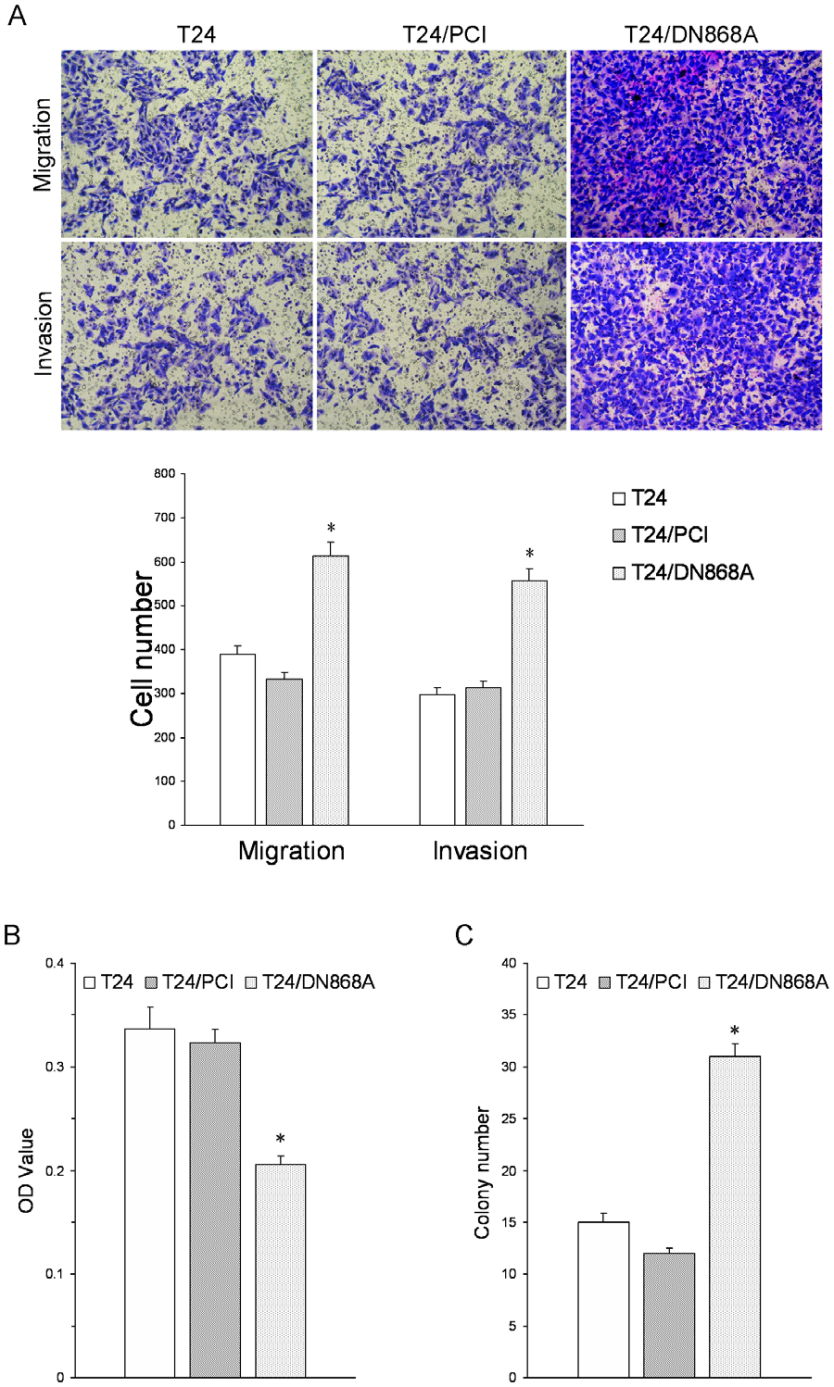
Progression capability of surviving T24/DN868A cells *in vitro*. A. Transwell assay for cell invasion and migration potential was performed. B. Adhesion assay for adhesive ability was determined. C. Soft agar colony formation assay was performed. (*P<0.05, compared with control cells).

To investigate the potential involvement of telomere maintenance and EMT in tumor development, we used surviving T24/DN868A cells as a model to examine their cellular behaviors after this conversion. Transwell assay results showed that both migration and invasive potential of surviving T24/DN868A cells were significantly increased compared with parental or control cells (Fig. 5A).

In adhesion assay, Matrigel was used to coat the 96-well plate to mimic the extracellular matrix (ECM). The O.D. value on behalf of adhesive cells demonstrated that the adhesive capacity of surviving T24/DN868A cell to Matrigel was markedly decreased (Fig. 5B).

We further explored the impact of this conversion on the clonogenicity of T24 cells. As expected, the soft agar assay demonstrated that surviving T24/DN868A cells prominently formed more and larger colonies than the parental T24 and control T24/PCI cells (Fig. 5C).

### Increased tumorigenesis of surviving T24/DN868A cells *in vivo*

**Figure 6:**
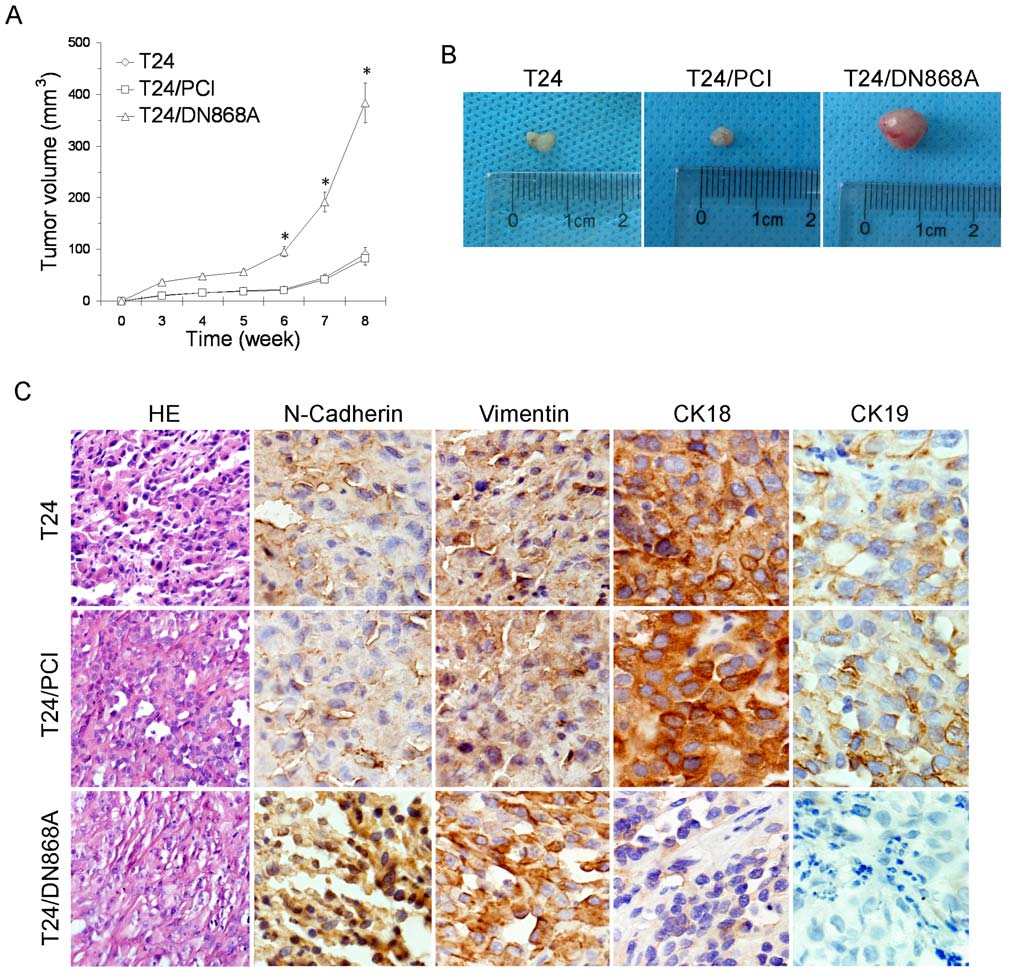
Tumorigenicity of surviving T24/DN868A cell in nude mice. A. Tumor growth assay. Cells were injected s.c. into the flank of nude mice and tumor volume was measured weekly until 8 weeks after injection (*P<0.001, compared to T24 and T24/PCI cells). B. Representative tumors isolated from T24, T24/PCI, and surviving T24/DN868A-injected nude mice. C. HE and immunohistochemical staining of EMT markers in tumors derived from T24, T24/PCI, and surviving T24/DN868A cells, respectively (40×).

It has been shown previously that the ALT pathway does not substitute for telomerase in the process of tumorigenesis *in vivo* [23]; therefore, we established the tumor xenograft by subcutaneous injection of 1×10^6^ T24, T24/PCI, or surviving T24/DN868A cells into 6–8-week-old nude mice (n = 4 mice per group). The tumor specimens were further analyzed by H&E staining. Tumors developed in all of nude mice 3–4 weeks after injection. Mice injected with surviving T24/DN868A cells showed a sharply accelerated speed in tumor formation (Fig. 6A) with a bigger mean volume of 383.5±51.08 mm^3^ after 8 weeks post injection, whereas the mean tumor volume in mice injected with T24 or T24/PCI cells were 90.3±12.89 and 82.6±10.07 mm^3^, respectively (Fig. 6B).

Histological staining showed that tumors derived from surviving T24/DN868A cells were cord-like and more aggressive while tumors derived from T24 or T24/PCI cells were rounded and less malignant (Fig. 6C). To further confirm that surviving T24/DN868A cells underwent EMT *in vivo*, immunohistochemical examination was performed in tumor specimens from nude mice. As the result, increased levels of N-cadherin and Vimentin in the nucleus and the cytoplasm as well as reduced levels of CK18 and CK19 between cell interfaces were observed in tissues derived from surviving T24/DN868A cells (Fig. 6C).

### Twist was activated to mediate EMT in surviving T24/DN868A cells

**Figure 7:**
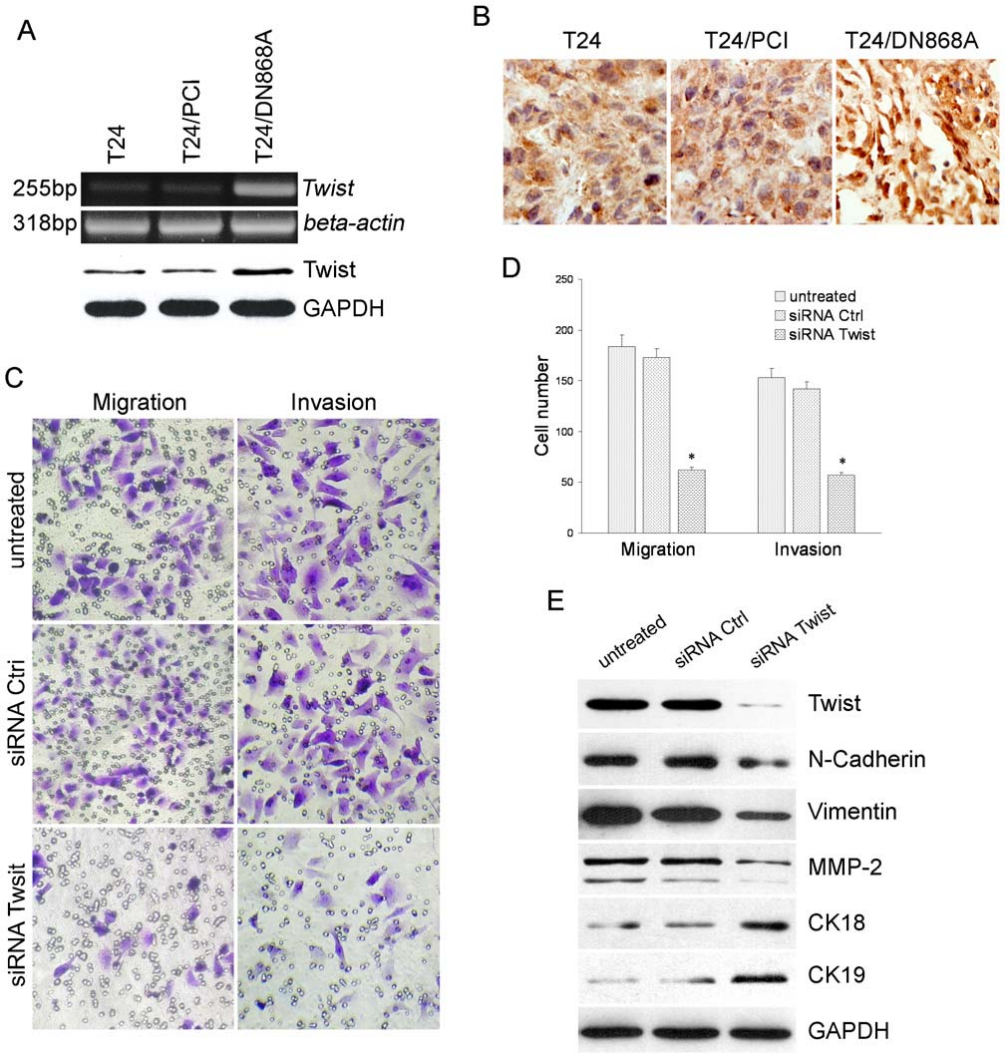
Twist mediated EMT in surviving T24/DN868A cells and tissues. A. RT-PCR and Western blot analysis of Twist expression in T24, T24/PCI, and surviving T24/DN868A cells. B. Detection of Twist expression by immunohistochemical staining in nude mice tumor samples. C and D. Surviving T24/DN868A cells were treated with siRNA specific to Twist, and analyzed for invasion and migration ability. E. Western blot analysis of EMT-associated proteins.

Next we wondered the molecular mechanism by which T24/DN868A conversion could modulate EMT. We examined many transcription factors such as Slug, Twist, Snail, Smad4 and ZEB1 by RT-PCR. The primer sequences for RT-PCR were shown in Table S1. As a result, only Slug and Twist were enhanced in surviving T24/DN868A cells, whereas the other transcription factors did not show obviously change (Fig. S1). Since Twist involves in promotion of EMT [24], and is capable to overcome oncogene-induced senescence in a variety of human cells [25], we focused on its alteration in surviving T24/DN868A cells. As a result, Twist was dramatically upregulated in these cells at both of mRNA and protein levels (Fig. 7A and Fig. S2). Furthermore, immunohistochemical staining of tissue samples exhibited a high percentage of Twist staining in surviving T24/DN868A cells. In addition to brownish dots representing Twist in the nucleus of a majority of cells, a number of cells also showed both cytoplasmic and nuclear staining (Fig. 7B). To further investigate the significance of Twist in this EMT process, we knocked down Twist with siRNA. Notably, down-modulation of Twist led to decreased capability of migration and invasion in surviving T24/DN868A cells (Fig. 7C and 7D). Meanwhile, these variations were concomitant with the elevation of CK18, CK19, and the suppression of N-cadherin, Vimentin, and MMP-2 in surviving T24/DN868A cells (Fig. 7E).

## Discussion

During the malignant progression of cancer cells, maintenance of telomere length is a crucial prerequisite for their immortalization [26], which is achieved by telomerase activity (TA) or alternative lengthening of telomeres (ALT) [27]. Inhibition of TA is considered as a potential target for cancer therapy; however, the situation is complicated by the existence of the ALT mechanism. Current understanding of telomere length kinetics in the absence of TA and subsequent engagement of the ALT pathway is primarily based on mismatched repair-deficient human colon cancer cells and laryngeal cancer cell line Hep-2 [28], [29]. In this work, DN hTERT was used to functionally inhibit TA in bladder cancer T24, 5637, and 253J cell lines, and sequenced alteration of telomere length was determined. Unfortunately, after transfection, most of these bladder cancer cell lines went to death, and no single cell clone could be cultured and passaged except for T24 cells. Our data show that although the proliferation rate of T24 cells with telomerase inhibition was retarded and the telomere length was shortened in earlier passage, the features associated with ALT-like pathway were observed during long-term culture. It is also worth noting that the total level of PML and MRN complex increased obviously, since more foci of MRE11, RAD50 and NBS1 were observed in the nucleus in surviving T24/DN868A cells (Fig. S3). While inhibiting telomerase resulted in either cell death or the reappearance of TA as reported previously [30], [31], our present study reveals no obvious up-regulation of telomerase at each passage based on the Q-FISH and TRF assay, suggesting that it is unlikely that remaining telomerase is the main cause for the observed telomere elongation in T24/DN868A cells. Instead, our data demonstrate that ALT-like mechanism could be the alternative way by which bladder cancer cells escape from telomerase inhibition.

Activation of ALT results from sets of genetic changes which are tumor-type specific, and many factors contribute to the selection of this mechanism for telomere maintenance. T24 cells express a mutant tumor suppressor protein p53 and a very low level of MSH6 [32], [33], which are considered as contributing factors for ALT activation [34], [35]. This may partly explain why among many bladder cancer cell lines, only T24 escaped from the cell crisis stage and continued to be alive.

Although ALT in tumors has not been studied extensively, it is now becoming clear that cells of epithelial origin maintain their telomeres via the telomerase pathway, whereas ALT is more frequently present in tumors of mesenchymal origin [5]. EMT is a process in which epithelial cells lose their epithelial phenotype and acquire new characteristic features of mesenchyme [36]. Interestingly, cell senescence can be bypassed during the activation of EMT that typically accompanies tumor progression [15]. In cataractous canine lens epithelial cells, the process of EMT also involves TERT [37]. In this study, we have noticed that the morphology of hTERT-inactivated T24 cells showed a gradual mesenchymal change from 25^th^ passage during the consistent culture as demonstrated by spindle shape and loss of intercellular junctions. Reduced epithelial markers and increased mesenchymal markers were displayed in both surviving T24/DN868A cells before the on set of ALT-like characteristics, and tumor samples derived from surviving cells, suggesting that EMT was activated to bypass cell senescence as seen in other publications (13, 15). Based on the different time of APBs appearance and EMT formation, very likely, the pathways leading to these changes may be different.

Telomerase and ALT are thought to be functionally inequitable in prompting tumor progression, but it remains contradictory which pathway is more aggressive. Some studies suggest that the ALT pathway, at least in some cell lines, does not functionally substitute for telomerase with respect to tumorigenicity and metastatic lesions [38]. In present study, we have examined the progression ability of surviving T24/DN868A cells. Compared with those of parental T24 or control cells, motility, invasion, and clone formation ability *in vitro* and tumorigenicity *in vivo* of surviving T24/DN868A cells were significantly enhanced, whereas adhesive ability of surviving T24/DN868A cells *in vitro* was inhibited, thus providing strong support that this fully malignant phenotype was triggered in surviving T24/DN868A cells with EMT.

The basic helix-loop-helix (bHLH) transcription factor Twist is a prompter of EMT [39], and its overexpression is significantly correlated with the stage and grade of human bladder tumor [40]. In the context of carcinogenesis, Twist can simultaneously suppress the senescence response and induce EMT [41]. In the present study, we found that Twist is overexpressed in surviving T24/DN868A cells from 24^th^ passage, and further aggregated in animal bladder tumor tissues. Consistently, depletion of Twist reduces the progression ability of surviving T24/DN868A and induces mesenchymal-to-epithelial (MET)-like change. Therefore, activation of EMT under telomerase inhibition requires the collaboration of the Twist-signaling pathway in bladder cancer.

Taken together, our data show that features associated with ALT-like mechanism and EMT could be induced after telomerase inhibition in bladder cancer cells with certain genetic background. Decreased adhesion ability *in vitro*, increased capabilities of migration, invasion, and colony formation *in vitro* and tumorigenesis *in vivo* of surviving T24/DN868A cells is associated with EMT induction, which is mediated by the activation of the Twist-signaling pathway. The progression of bladder cancer with ALT-like pathway after telomerase inhibition and the activation of EMT suggest a novel possible mechanism of drug resistance to anti-telomerase therapy in clinic bladder cancer patients.

## Supporting Information

Figure S1 Expression of transcriptional factors was detected by RT-PCR. ß-actin was used as an internal standard. Three experiments were performed independently.
(TIF)

Figure S2 Expression of Twist in different passage of T24/DN868A was detected by Western blot. GAPDH was used as a loading control.
(TIF)

Figure S3 MRN status in surviving T24/DN868A cells. Double immunofluorescence analysis with mouse anti-PML and rabbit anti-MRE11, RAD50 or NBS1 in surviving T24/DN868A cells was shown. Red spots represent MRN components, green spots represent PML, and yellow sparkles indicate the colocalization of PML with MRN. Accumulated MRN spots in the nucleus were observed in surviving T24/DN868A cells, and most of them were colocalized with PML sparkles (60×).
(TIF)

Table S1 The primer sequences for RT-PCR.
(DOC)
