# Supplementary figures and images for: Twisted Epithelial-to-Mesenchymal Transition Promotes Progression of Surviving Bladder Cancer T24 Cells with hTERT-Dysfunction
Source: PLoS One. 2011 Nov 15;6(11):e27748. doi: 10.1371/journal.pone.0027748 (PMC3216997; doi:10.1371/journal.pone.0027748)

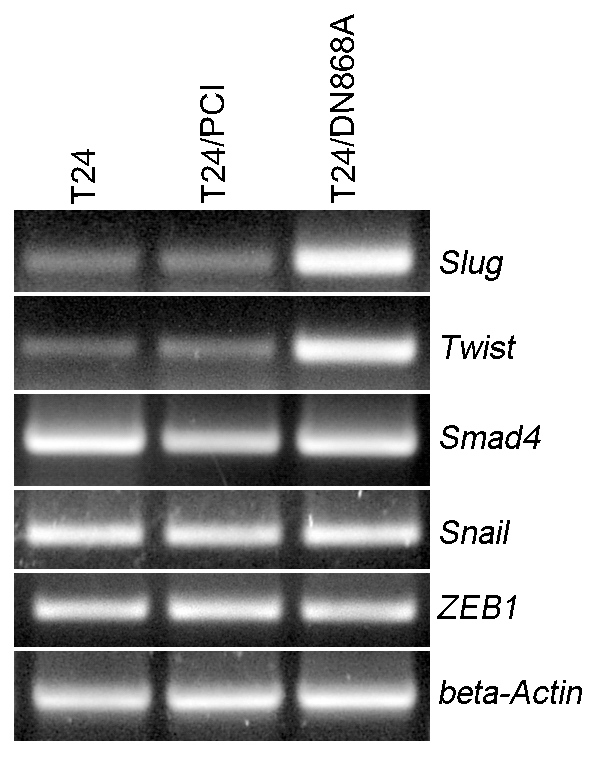

Supplement: Figure S1 — Expression of transcriptional factors was detected by RT-PCR. ß-actin was used as an internal standard. Three experiments were performed independently. (TIF) [file pone.0027748.s001.tif]

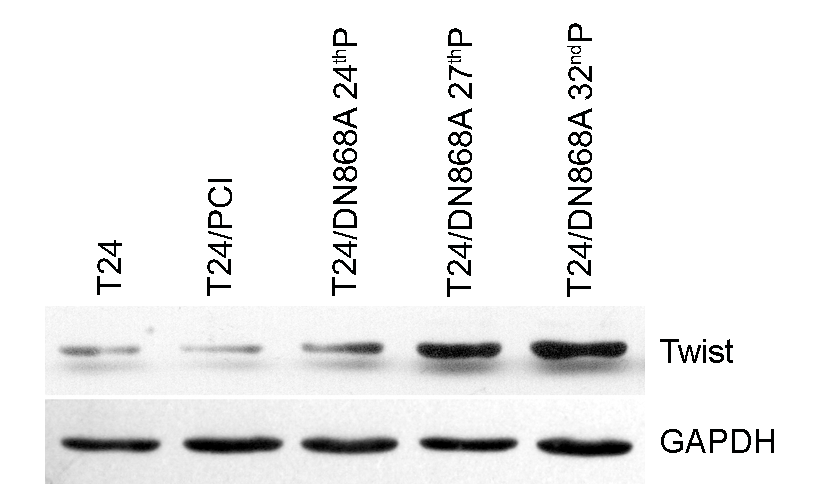

Supplement: Figure S2 — Expression of Twist in different passage of T24/DN868A was detected by Western blot. GAPDH was used as a loading control. (TIF) [file pone.0027748.s002.tif]

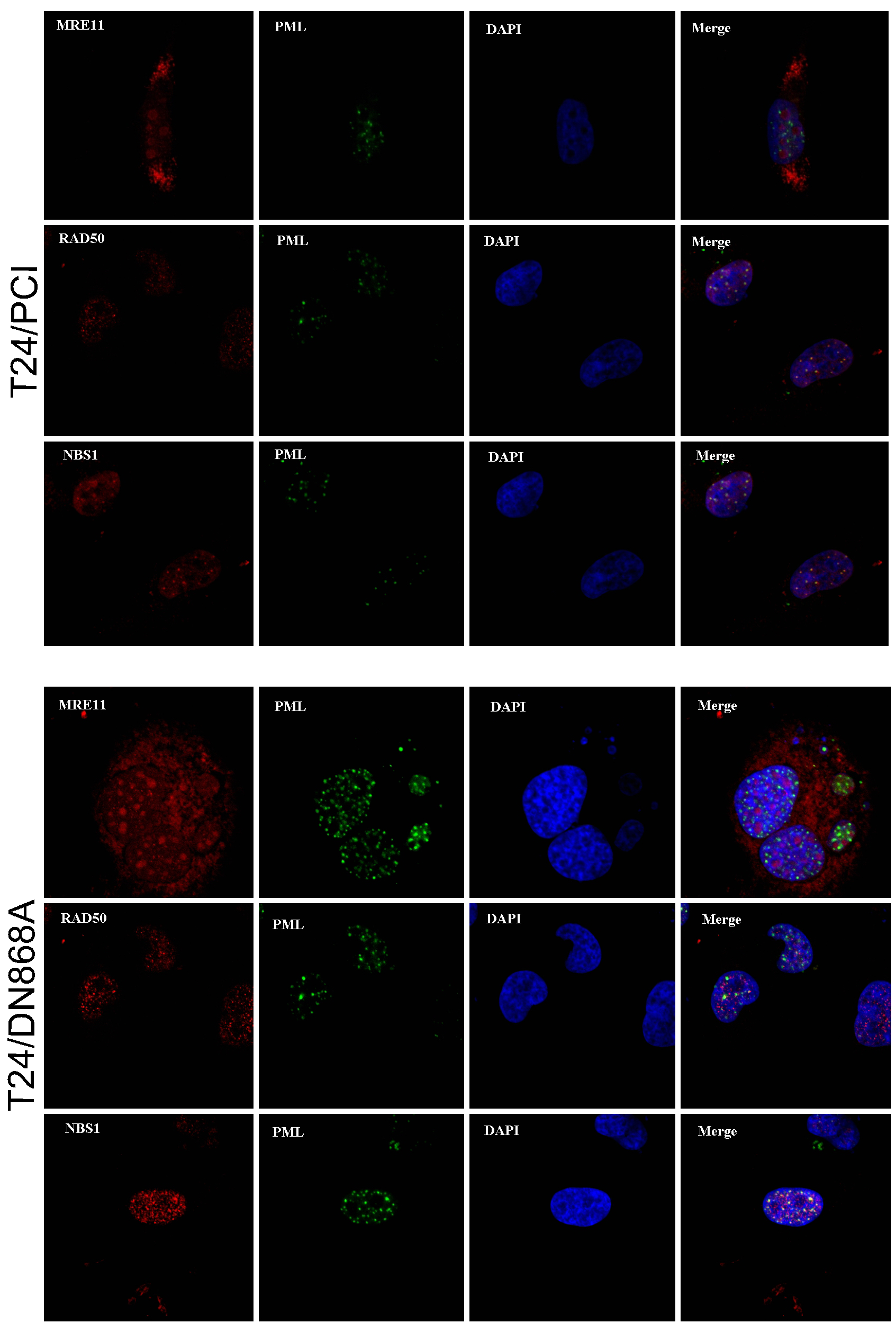

Supplement: Figure S3 — MRN status in surviving T24/DN868A cells. Double immunofluorescence analysis with mouse anti-PML and rabbit anti-MRE11, RAD50 or NBS1 in surviving T24/DN868A cells was shown. Red spots represent MRN components, green spots represent PML, and yellow sparkles indicate the colocalization of PML with MRN. Accumulated MRN spots in the nucleus were observed in surviving T24/DN868A cells, and most of them were colocalized with PML sparkles (60×). (TIF) [file pone.0027748.s003.tif]
