# Supplementary material for: Twisted Epithelial-to-Mesenchymal Transition Promotes Progression of Surviving Bladder Cancer T24 Cells with hTERT-Dysfunction
Source: PLoS One. 2011 Nov 15;6(11):e27748. doi: 10.1371/journal.pone.0027748 (PMC3216997; doi:10.1371/journal.pone.0027748)
Supplement: Table S1 — The primer sequences for RT-PCR. (DOC) [file pone.0027748.s004.doc]

**Supplementary Materials and Methods**

*Reverse Transcription-PCR*

Total cellular RNA was isolated using TRIzol reagent (Invitrogen, Carlsbad, CA). One microgram of RNA was reverse transcribed using a ReverAid first strand cDNA synthesis kit (Fermentas, Vilnius, Lithuania, EU). All of the PCR reactions were initiated with incubation at 94°C for 2min, followed by 30 cycles of 94°C, 30s; 59~61°C, 30s; and 72°C, 2min. Reactions were finished with a 72°C, 7min extension. The sequence of the primers was displayed in Table 1.

*Double Immunofluorescence*

Cells were grown on glass coverslips and fixed in 4% paraformaldehyde (PFA). After washing with PBS, the cells were permeabilized using 0.1% Triton X-100 solution and blocked with 10% donkey serum. Following incubation with the primary antibodies PML (mouse) and MRE11, RAD50 or NBS1(Rabbit), they were incubated with the fluorescently labeled secondary antibodies TRITC-anti-rabbit or FITC-anti-mouse (invitrogen). Images were collected under confocal microscopy and processed using Adobe Photoshop 7.0.

**Table 1**

The primer sequences for RT-PCR

| Gene | Primer sequence | Product (bp) |
| --- | --- | --- |
| Twist1 | F5’-CGGACAAGCTGAGCAAGATT-3’ | 255 |
|  | R5’-CCTTCTCTGGAAACAATGAC-3’ |  |
| Slug | F5’-TCCTGGTCAAGAAGCATTTCAACG-3’ | 184 |
|  | R5’-TGGAATGGAGCAGCGGTAGTC-3’ |  |
| ZEB1 | F5’-TGGCATACACCTACTCAACTACGG-3’ | 689 |
|  | R5’-TTCCTCCTCCTCCTCCTCTTCC-3’ |  |
| Snail | F5’-GCCTTCAACTGCAAATACTGC-3’ | 249 |
|  | R5’-CTTCTTGACATCTGAGTGGGTC-3’ |  |
| Smad4 | F5’-CCTGAACTGTTTGTACCTCTGGGCCATATTGC-3’ | 243 |
|  | R5’-CAAATTTCTGAAGAGTAGGTGATCCGGGTGGAG-3’ |  |
| β-actin | F5’-ATCATGTTTGAGACCTTCAACA-3’ | 318 |
|  | R5’-CATCTCTTGCTCGAAGTCCA-3’ |  |
